# Supplementary material for: Retinal stem cells modulate proliferative parameters to coordinate post-embryonic morphogenesis in the eye of fish
Source: eLife. 2019 Mar 26;8:e42646. doi: 10.7554/eLife.42646 (PMC6486154; doi:10.7554/eLife.42646)
Supplement: Supplementary file 3. — Step by step instructions on how to open Supplementary file 1 and Supplementary file 2. [file elife-42646-supp3.pdf]

Instructions for using the supplementary model files

The model used in this work has been created in the Multi-Scale Modeling and Simulation Platform EPISIM, which can be downloaded at:

http://tigacenter.bioquant.uni-heidelberg.de/episim.html

EPISIM consists of two software components: EPISIM Modeller and EPISIM Simulator.

Importing the cell behavioral model project into EPISIM Modeller

In EPISIM, the cell behavioral model represents the internal logic of each individual cell. To use a metaphor, it's the "genetic code", which during the simulation will result in context-dependent "expression" of selected submodels.

- Open EPISIM Modeller
- Click on "File > Import"
- Select "EPISIM Modeller Project", then click "Next"
- Select the option "Select archive file", then click "Browse…"
- Navigate to the folder with the file "Tsingos_Fish_Eye_Model.zip" and select it.
- Click "Finish".
- The cell behavioral model will now be loaded into the workspace.

Executing the simulation in EPISIM Simulator

EPISIM Simulator integrates the cell behavioral model with the hard-coded biomechanical model (which includes *e.g.* definitions for the initial condition, geometry, and the force-balance calculation), and executes the simulation while providing visualization.

- Open EPISIM Simulator
- Click on "File > Open EPISIM-Cell-Model"
- Navigate to the folder with the file "Tsingos_fish_eye_model.jar" and select it.
- To change cell behavioral model parameters, use the tab "Cell-Behavioral-Model"
- To change biomechanical model parameters, use the tab "Biomechanical-Model"
- Click on the play icon (bottom left triangle) to start the simulation.
